# 2-(2-Chloro­phen­yl)acetic acid

**DOI:** 10.1107/S1600536812023938

**Published:** 2012-05-31

**Authors:** Rajni Kant, Vivek K. Gupta, Kamini Kapoor, B. Narayana

**Affiliations:** a X-ray Crystallography Laboratory, Post-Graduate Department of Physics & Electronics, University of Jammu, Jammu Tawi 180 006, India; b Department of Studies in Chemistry, Mangalore University, Mangalagangotri 574 199, India

## Abstract

In the title compound, C_8_H_7_ClO_2_, the carboxyl group forms a dihedral angle of 74.83 (9)° with the benzene ring plane. In the crystal, mol­ecules are linked into inversion dimers by pairs of O—H⋯O hydrogen bonds. The dimers are linked into layers parallel to the *bc* plane by weak C—H⋯O inter­actions.

## Related literature

For applications of phenyl­acetic acids, see: Castellari & Ottani (1995 ▶); Deshpande *et al.* (2008 ▶); Hata *et al.* (1986 ▶). For the crystal structure of isostructural 2-(2-bromo­phen­yl)acetic acid, see: Kant *et al.* (2012 ▶).
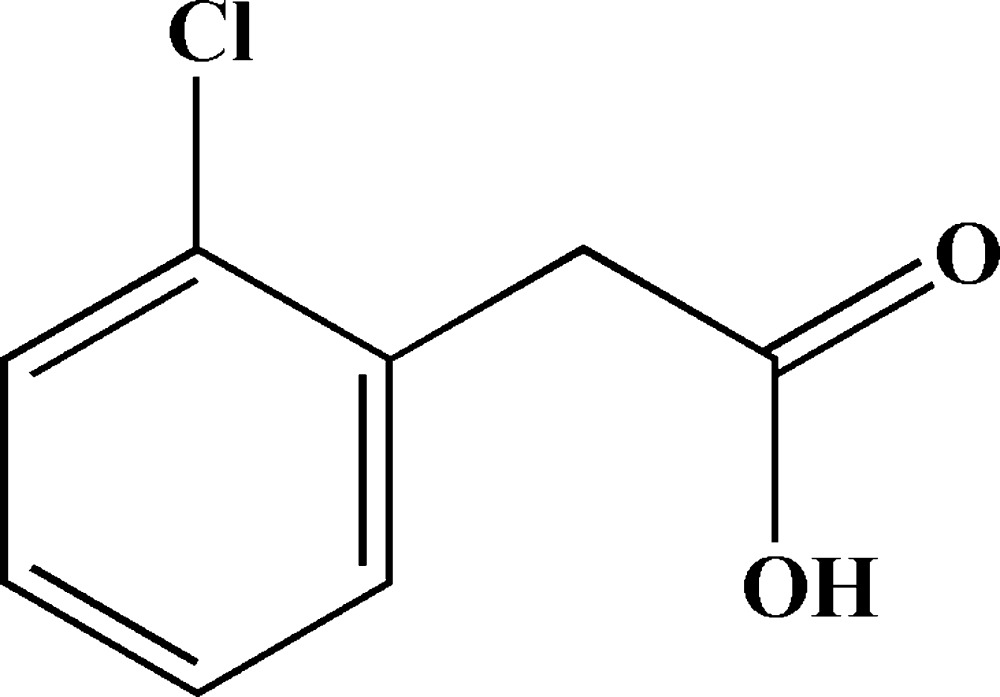


## Experimental

### 

#### Crystal data

C_8_H_7_ClO_2_
*M* *_r_* = 170.59
Monoclinic, 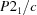

*a* = 9.1473 (7) Å
*b* = 5.8265 (3) Å
*c* = 15.4299 (7) Å
β = 101.155 (5)°
*V* = 806.83 (8) Å^3^
*Z* = 4
Mo *K*α radiation
μ = 0.42 mm^−1^
*T* = 293 K
0.3 × 0.2 × 0.2 mm

#### Data collection

Oxford Diffraction Xcalibur Sapphire3 diffractometer
Absorption correction: multi-scan (*CrysAlis PRO*; Oxford Diffraction, 2010 ▶) *T*_min_ = 0.748, *T*_max_ = 1.000
9367 measured reflections
1583 independent reflections
1173 reflections with *I* > 2σ(*I*)
*R*_int_ = 0.045

#### Refinement

*R*[*F* ^2^ > 2σ(*F* ^2^)] = 0.052
*wR*(*F* ^2^) = 0.145
*S* = 1.06
1583 reflections
100 parameters
H-atom parameters constrained
Δρ_max_ = 0.30 e Å^−3^
Δρ_min_ = −0.33 e Å^−3^

### 

Data collection: *CrysAlis PRO* (Oxford Diffraction, 2010 ▶); cell refinement: *CrysAlis PRO*; data reduction: *CrysAlis PRO*; program(s) used to solve structure: *SHELXS97* (Sheldrick, 2008 ▶); program(s) used to refine structure: *SHELXL97* (Sheldrick, 2008 ▶); molecular graphics: *ORTEP-3 for Windows* (Farrugia, 1997 ▶); software used to prepare material for publication: *PLATON* (Spek, 2009 ▶).

## Supplementary Material

Crystal structure: contains datablock(s) I, New_Global_Publ_Block. DOI: 10.1107/S1600536812023938/gk2495sup1.cif

Structure factors: contains datablock(s) I. DOI: 10.1107/S1600536812023938/gk2495Isup2.hkl

Supplementary material file. DOI: 10.1107/S1600536812023938/gk2495Isup3.cml

Additional supplementary materials:  crystallographic information; 3D view; checkCIF report

## Figures and Tables

**Table 1:** Hydrogen-bond geometry (Å, °)

*D*—H⋯*A*	*D*—H	H⋯*A*	*D*⋯*A*	*D*—H⋯*A*
O10—H10⋯O9^i^	0.82	1.82	2.639 (4)	173
C6—H6⋯O9^ii^	0.93	2.57	3.469 (4)	163

Symmetry codes: (i) 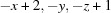; (ii) 
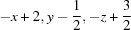
.

**Fig. 1.**
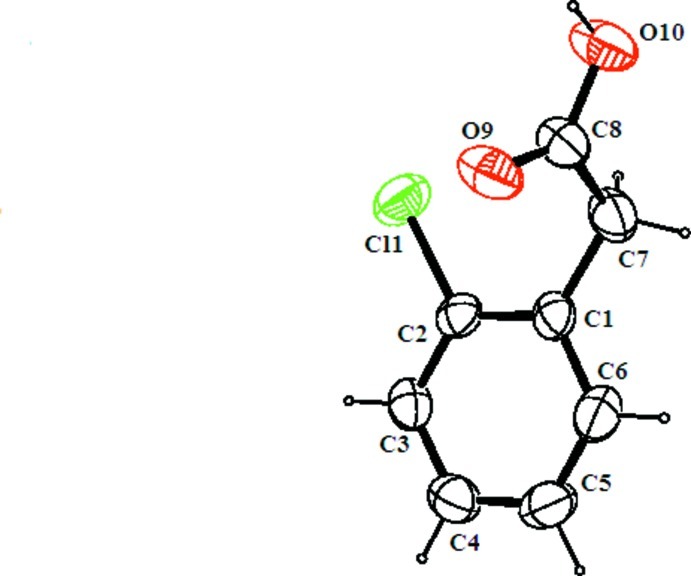
ORTEP view of the molecule with displacement ellipsoids are drawn at the 40% probability level. H atoms are shown as small spheres of arbitrary radii.

**Fig. 2.**
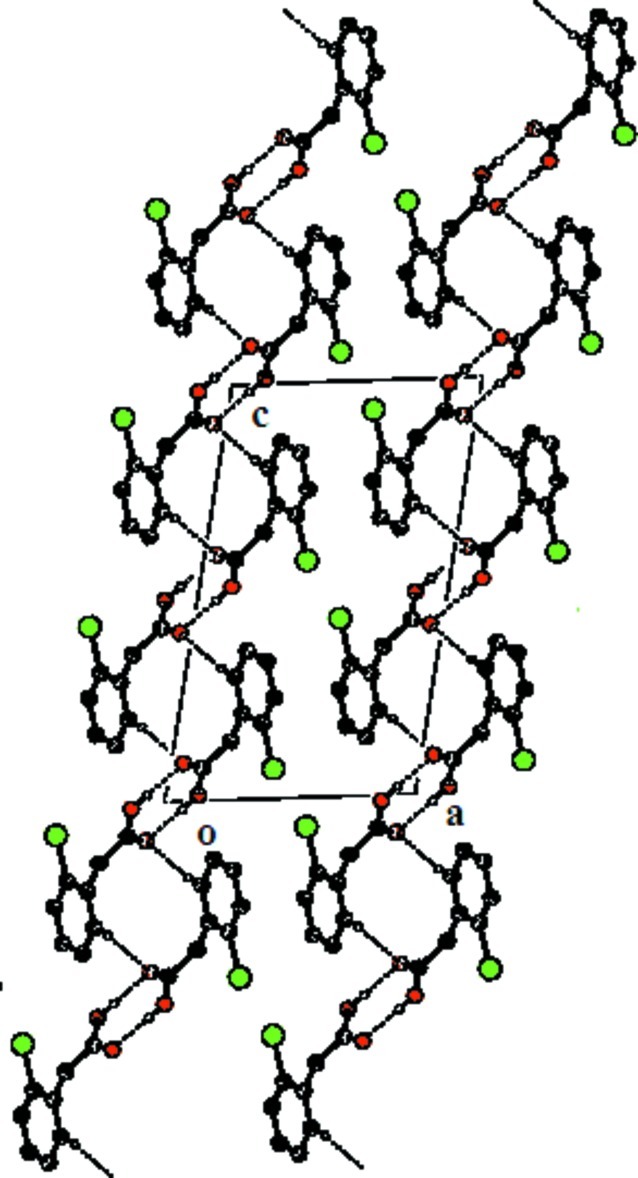
Projection of the crystal packing along the b axis. Hydrogen atoms are omitted for clarity and hydrogen bonds are shown with dashed lines.

## supplementary crystallographic information

### Comment

Derivatives of phenyl acetic acids are used as ingredients in perfume to provide honey like odour, for the preparation of a nonsteroidal anti-inflammatory drug like diclofenac (Hata *et al.*, 1986; Castellari & Ottani, 1995), and as intermediate compounds for the synthesis of heterocyclic compounds (Deshpande *et al.*, 2008). In continuation of our work on substituted phenylacetic acid (Kant *et al.*, 2012), we report the crystal structure of a new derivative, 2-(2-chlorophenyl)acetic acid (I). The title compound is closely isostructural with its bromo analogue (Kant *et al.*, 2012).

### Experimental

The title compound was purchased from the Spectrochem Ltd. and single crystal was grown from ethyl acetate and toluene (1:1) mixture by slow evaporation method (m.p. 366–369 K).

### Refinement

All H atoms were positioned geometrically and were treated as riding on their parent atoms, with O—H distances of 0.82 Å and C—H distances of 0.93–0.97 Å and with *U*_iso_(H) = 1.2*U*_eq_(C/O).

### Crystal data

**Table:**

C_8_H_7_ClO_2_	*F*(000) = 352
*M**_r_* = 170.59	*D*_x_ = 1.404 Mg m^−^^3^
Monoclinic, *P*2_1_/*c*	Melting point = 369–366 K
Hall symbol: -P 2ybc	Mo *K*α radiation, λ = 0.71073 Å
*a* = 9.1473 (7) Å	Cell parameters from 3981 reflections
*b* = 5.8265 (3) Å	θ = 3.5–29.1°
*c* = 15.4299 (7) Å	µ = 0.42 mm^−^^1^
β = 101.155 (5)°	*T* = 293 K
*V* = 806.83 (8) Å^3^	Block, colourless
*Z* = 4	0.3 × 0.2 × 0.2 mm

### Data collection

**Table:**

Oxford Diffraction Xcalibur Sapphire3 diffractometer	1583 independent reflections
Radiation source: fine-focus sealed tube	1173 reflections with *I* > 2σ(*I*)
Graphite monochromator	*R*_int_ = 0.045
Detector resolution: 16.1049 pixels mm^-1^	θ_max_ = 26.0°, θ_min_ = 3.8°
ω scans	*h* = −11→11
Absorption correction: multi-scan (*CrysAlis PRO*; Oxford Diffraction, 2010)	*k* = −7→7
*T*_min_ = 0.748, *T*_max_ = 1.000	*l* = −19→19
9367 measured reflections	

### Refinement

**Table:**

Refinement on *F*^2^	Primary atom site location: structure-invariant direct methods
Least-squares matrix: full	Secondary atom site location: difference Fourier map
*R*[*F*^2^ > 2σ(*F*^2^)] = 0.052	Hydrogen site location: inferred from neighbouring sites
*wR*(*F*^2^) = 0.145	H-atom parameters constrained
*S* = 1.06	*w* = 1/[σ^2^(*F*_o_^2^) + (0.0608*P*)^2^ + 0.4607*P*] where *P* = (*F*_o_^2^ + 2*F*_c_^2^)/3
1583 reflections	(Δ/σ)_max_ = 0.001
100 parameters	Δρ_max_ = 0.30 e Å^−^^3^
0 restraints	Δρ_min_ = −0.33 e Å^−^^3^

### Special details

**Table:**

Experimental. *CrysAlis PRO*, Oxford Diffraction Ltd., Version 1.171.34.40 (release 27–08-2010 CrysAlis171. NET) (compiled Aug 27 2010,11:50:40) Empirical absorption correction using spherical harmonics, implemented in SCALE3 ABSPACK scaling algorithm.
Geometry. All e.s.d.'s (except the e.s.d. in the dihedral angle between two l.s. planes) are estimated using the full covariance matrix. The cell e.s.d.'s are taken into account individually in the estimation of e.s.d.'s in distances, angles and torsion angles; correlations between e.s.d.'s in cell parameters are only used when they are defined by crystal symmetry. An approximate (isotropic) treatment of cell e.s.d.'s is used for estimating e.s.d.'s involving l.s. planes.
Refinement. Refinement of *F*^2^ against ALL reflections. The weighted *R*-factor *wR* and goodness of fit *S* are based on *F*^2^, conventional *R*-factors *R* are based on *F*, with *F* set to zero for negative *F*^2^. The threshold expression of *F*^2^ > σ(*F*^2^) is used only for calculating *R*-factors(gt) *etc*. and is not relevant to the choice of reflections for refinement. *R*-factors based on *F*^2^ are statistically about twice as large as those based on *F*, and *R*- factors based on ALL data will be even larger.

### Fractional atomic coordinates and isotropic or equivalent isotropic displacement parameters (Å^2^)

**Table:**

	*x*	*y*	*z*	*U*_iso_*/*U*_eq_	
Cl1	0.58539 (10)	0.25918 (15)	0.57120 (5)	0.0721 (3)	
C1	0.7450 (3)	0.0180 (4)	0.70745 (16)	0.0451 (6)	
C2	0.6591 (3)	0.2111 (4)	0.68252 (16)	0.0447 (6)	
C3	0.6269 (3)	0.3678 (5)	0.74270 (18)	0.0516 (7)	
H3	0.5691	0.4962	0.7238	0.062*	
C4	0.6811 (4)	0.3324 (5)	0.8313 (2)	0.0599 (8)	
H4	0.6599	0.4369	0.8727	0.072*	
C5	0.7665 (4)	0.1427 (6)	0.85832 (19)	0.0657 (9)	
H5	0.8028	0.1180	0.9182	0.079*	
C6	0.7986 (4)	−0.0111 (5)	0.79697 (19)	0.0602 (8)	
H6	0.8577	−0.1380	0.8162	0.072*	
C7	0.7754 (4)	−0.1586 (5)	0.6417 (2)	0.0604 (8)	
H7A	0.8219	−0.2908	0.6740	0.073*	
H7B	0.6808	−0.2086	0.6070	0.073*	
C8	0.8723 (3)	−0.0803 (5)	0.57996 (18)	0.0527 (7)	
O9	0.9496 (2)	0.0930 (4)	0.59199 (13)	0.0672 (6)	
O10	0.8700 (3)	−0.2177 (4)	0.51387 (14)	0.0729 (7)	
H10	0.9245	−0.1674	0.4820	0.109*	

### Atomic displacement parameters (Å^2^)

**Table:**

	*U*^11^	*U*^22^	*U*^33^	*U*^12^	*U*^13^	*U*^23^
Cl1	0.0835 (7)	0.0807 (6)	0.0476 (4)	−0.0019 (4)	0.0015 (4)	0.0124 (4)
C1	0.0496 (16)	0.0395 (13)	0.0501 (14)	−0.0030 (11)	0.0193 (12)	0.0039 (11)
C2	0.0466 (15)	0.0484 (15)	0.0403 (12)	−0.0089 (12)	0.0117 (11)	0.0049 (11)
C3	0.0537 (17)	0.0421 (14)	0.0629 (16)	0.0005 (12)	0.0206 (13)	0.0032 (13)
C4	0.070 (2)	0.0558 (17)	0.0571 (17)	−0.0072 (15)	0.0218 (15)	−0.0096 (14)
C5	0.076 (2)	0.077 (2)	0.0423 (14)	−0.0015 (18)	0.0081 (14)	0.0044 (15)
C6	0.065 (2)	0.0565 (17)	0.0597 (17)	0.0089 (15)	0.0142 (14)	0.0149 (14)
C7	0.076 (2)	0.0430 (15)	0.0692 (18)	−0.0054 (15)	0.0318 (16)	−0.0039 (14)
C8	0.0549 (17)	0.0486 (15)	0.0576 (16)	−0.0054 (14)	0.0184 (13)	−0.0096 (13)
O9	0.0748 (15)	0.0647 (13)	0.0710 (13)	−0.0229 (12)	0.0362 (11)	−0.0244 (11)
O10	0.0876 (17)	0.0699 (14)	0.0708 (14)	−0.0292 (12)	0.0393 (12)	−0.0291 (11)

### Geometric parameters (Å, º)

**Table:**

Cl1—C2	1.742 (3)	C5—C6	1.376 (4)
C1—C2	1.384 (4)	C5—H5	0.9300
C1—C6	1.384 (4)	C6—H6	0.9300
C1—C7	1.508 (4)	C7—C8	1.492 (4)
C2—C3	1.374 (4)	C7—H7A	0.9700
C3—C4	1.377 (4)	C7—H7B	0.9700
C3—H3	0.9300	C8—O9	1.227 (3)
C4—C5	1.370 (5)	C8—O10	1.293 (3)
C4—H4	0.9300	O10—H10	0.8200
			
C2—C1—C6	116.7 (2)	C6—C5—H5	120.0
C2—C1—C7	122.4 (2)	C5—C6—C1	121.7 (3)
C6—C1—C7	120.8 (3)	C5—C6—H6	119.1
C3—C2—C1	122.5 (2)	C1—C6—H6	119.1
C3—C2—Cl1	117.8 (2)	C8—C7—C1	115.5 (2)
C1—C2—Cl1	119.7 (2)	C8—C7—H7A	108.4
C2—C3—C4	119.2 (3)	C1—C7—H7A	108.4
C2—C3—H3	120.4	C8—C7—H7B	108.4
C4—C3—H3	120.4	C1—C7—H7B	108.4
C5—C4—C3	119.8 (3)	H7A—C7—H7B	107.5
C5—C4—H4	120.1	O9—C8—O10	123.3 (3)
C3—C4—H4	120.1	O9—C8—C7	123.5 (2)
C4—C5—C6	120.1 (3)	O10—C8—C7	113.2 (2)
C4—C5—H5	120.0	C8—O10—H10	109.5
			
C6—C1—C2—C3	−0.2 (4)	C4—C5—C6—C1	−0.9 (5)
C7—C1—C2—C3	177.6 (3)	C2—C1—C6—C5	0.8 (4)
C6—C1—C2—Cl1	−179.2 (2)	C7—C1—C6—C5	−177.0 (3)
C7—C1—C2—Cl1	−1.4 (4)	C2—C1—C7—C8	68.3 (4)
C1—C2—C3—C4	−0.3 (4)	C6—C1—C7—C8	−114.0 (3)
Cl1—C2—C3—C4	178.8 (2)	C1—C7—C8—O9	15.7 (5)
C2—C3—C4—C5	0.2 (4)	C1—C7—C8—O10	−166.3 (3)
C3—C4—C5—C6	0.4 (5)		

### Hydrogen-bond geometry (Å, º)

**Table:**

*D*—H···*A*	*D*—H	H···*A*	*D*···*A*	*D*—H···*A*
O10—H10···O9^i^	0.82	1.82	2.639 (4)	173
C6—H6···O9^ii^	0.93	2.57	3.469 (4)	163

Symmetry codes: (i) −*x*+2, −*y*, −*z*+1; (ii) −*x*+2, *y*−1/2, −*z*+3/2.

## References

[bb1] Castellari, C. & Ottani, S. (1995). *Acta Cryst.* C**51**, 2612–2615.

[bb2] Deshpande, P. P., Nanduri, V. B., Pullockaran, A., Christie, H., Mueller, R. H. & Patel, R. N. (2008). *J. Ind. Microbiol. Biotechnol.* **35**, 901–906. 10.1007/s10295-008-0363-4 18496722

[bb3] Farrugia, L. J. (1997). *J. Appl. Cryst.* **30**, 565.

[bb4] Hata, T., Sato, S. & Tamura, C. (1986). *Acta Cryst.* C**42**, 452–454.

[bb5] Kant, R., Kapoor, K. & Narayana, B. (2012). *Acta Cryst.* E**68**, o1704. 10.1107/S1600536812020545 PMC3379297 22719495

[bb6] Oxford Diffraction (2010). *CrysAlis PRO* Oxford Diffraction Ltd, Yarnton, England.

[bb7] Sheldrick, G. M. (2008). *Acta Cryst.* A**64**, 112–122. 10.1107/S0108767307043930 18156677

[bb8] Spek, A. L. (2009). *Acta Cryst.* D**65**, 148–155. 10.1107/S090744490804362X PMC2631630 19171970

